# Antibodies against Small Ubiquitin-like Modifier Activating Enzyme May Be a Protective Factor from Rapid Progressive Interstitial Lung Disease in Patients Bearing Antibodies against Melanoma Differentiation Associated Gene 5

**DOI:** 10.3390/jcm13030725

**Published:** 2024-01-26

**Authors:** Hung-Cheng Tsai, Wei-Sheng Chen, Yi-Syuan Sun, Chien-Chih Lai, Ying-Ying Yang, Wen-Ru Chou, Hsien-Tzung Liao, Chang-Youh Tsai, Chung-Tei Chou

**Affiliations:** 1 Division of Allergy, Immunology & Rheumatology, Taipei Veterans General Hospital, Taipei 112, Taiwan; hctsai7@vghtpe.gov.tw (H.-C.T.); wschen2@vghtpe.gov.tw (W.-S.C.); yssun2@vghtpe.gov.tw (Y.-S.S.); cclai3@vghtpe.gov.tw (C.-C.L.); ctchou@vghtpe.gov.tw (C.-T.C.); 2 Faculty of Medicine, School of Medicine, National Yang Ming Chiao Tung University, Taipei 11267, Taiwan; yangyy@vghtpe.gov.tw; 3 Institute of Clinical Medicine, National Yang-Ming Chiao Tung University, Taipei Campus, Taipei 112, Taiwan; 4 Division of Clinical Skills Training Center, Department of Medical Education, Taipei Veterans General Hospital, Taipei 112, Taiwan; 5 Department of Medical Education, Taipei Veterans General Hospital, Taipei 112, Taiwan; 6 Division of Chest Medicine, Department of Medicine, Fu Jen Catholic University Hospital, 69 Guitz Rd., New Taipei City 24352, Taiwan; chouwenru@gmail.com; 7 School of Medicine, Fu Jen Catholic University, New Taipei City 242, Taiwan; 8 Division of Immunology & Rheumatology, Department of Medicine, Fu Jen Catholic University Hospital, 69 Guitz Rd., New Taipei City 24352, Taiwan

**Keywords:** anti-MDA5 antibodies, dermatomyositis (DM), anti-SAE antibodies, rapidly progressive interstitial lung disease (RPILD)

## Abstract

**Background:** Anti-MDA5 antibody-bearing (anti-MDA5^+^)-dermatomyositis (DM) or polymyositis (PM) is notorious for causing rapidly progressive interstitial lung disease (RPILD) and/or cancers with high mortality rate. However, anti-MDA5 antibodies (Abs) are also found in other connective tissue diseases and their link with RPILD, especially with regard to the mortality rate, are unknown. **Methods:** We retrospectively recruited 71 patients bearing anti-MDA5-Abs in serum, stratified them in terms of a presence or absence of RPILD, and evaluated their clinical features, laboratory findings, associated myositis antibodies, concurrent connective tissue disease (CTD) as well as newly developed malignancies. **Results:** In total, 39 (55%) patients presented with DM/PM, but 32 (45%) did not. In total, 22 of the former and 11 of the latter developed RPILD eventually, accounting for a total of 46% of all MDA-5 bearing patients. On the other hand, 15 of all 71 (21.1%) patients had cancers. Among the 32 patients who did not have DM/PM, 27 (38.0% of all 71) had other CTDs, indicating that only 5 (7.0% of 71) patients did not have CTDs. Senility (odds ratio (OR) = 1.816, *p* = 0.032), presence of anti-Ro-52 antibody (OR = 1.676, *p* = 0.018), elevated C-reactive protein (CRP, OR = 4.354, *p* < 0.001) and carcinoembryonic antigen (CEA, OR = 2.625, *p* = 0.005) posed risks for RPILD. High lactose dehydrogenase (LDH, *p* = 0.009), CRP (*p* = 0.001) and CEA (*p* = 0.001), ferritin (*p* ≤ 0.001) and low albumin (*p* ≤ 0.001) were significantly associated with mortality. Anti-SAE antibodies were negatively correlated with RPILD as analyzed by univariate (OR = 0.245, *p* = 0.017) and multivariate (OR = 0.058, *p* = 0.036) regressions, indicating that they may be a protective factor in relation to RPILD (OR = 0.543, *p* = 0.008) or fatality (OR = 0.707, *p* = 0.012), which was also demonstrated in subgroup analyses. **Conclusions:** In contrast to various risk factors for RPILD or mortality, anti-SAE antibodies might conversely be a protective factor in anti-MDA5^+^ patients.

## 1. Introduction

Amyopathic dermatomyositis (ADM) is defined as a disorder with typical cutaneous manifestations of dermatomyositis (DM), but without evidence of muscle involvement [1]. A subset of ADM with anti-melanoma differentiation-associated gene 5 antibodies (anti-MDA5 Abs) has been especially linked with rapidly progressive interstitial lung disease (RPILD) with very poor prognosis in eastern Asian patients [2,3,4,5]. The prevalence of RPILD in anti-MDA5^+^-DM patients ranges from 38% to 70% [6,7,8]. The six-month survival rate of RPILD-ADM patients can be as low as 41% despite aggressive treatments [9].

Various poor prognostic factors in MDA5^+^-DM with ILD have been identified, including older age at disease onset, skin involvement, higher levels of lactose dehydrogenase (LDH), erythrocyte sedimentation rate (ESR), C-reactive protein (CRP), ferritin, interleukin (IL)-6, and serum tumor markers, etc. [10,11]. However, the etiologies of anti-MDA5-associated RPILD have not been fully characterized. Only sporadic studies have pointed out the link between viral infection and anti-MDA5^+^-DM-associated RPILD [12,13,14].

On the other hand, not only RPILD, but also connective tissue disease-associated interstitial lung diseases (CTD-ILDs), including those associated with myositis, will cause progressive fibrosing ILD (PF-ILD). A diagnosis of PF-ILD must depend on the development of at least two of the three features within the previous one year without explainable reasons. These three features are a worsening of the respiratory symptoms, physiological evidence of disease progression, and radiological evidence of disease progression [15]. Despite cumulating evidence in favor of the efficacy of anti-fibrotic drugs such as nintedanib on PF-ILD [16], there is still a lack of trials exploring the effect of such drugs on RPILD.

DM patients with anti-small ubiquitin-like modifier activating enzyme (anti-SAE) Abs tend to develop severe skin diseases without overt muscle involvement [17]. Anti-Ro52 Abs have been linked to a higher prevalence of RPILD with a poorer prognosis in the anti-MDA5^+^DM cohort [18].

Before now, there have been no specific criteria raised for ADM. Both 2017 EULAR/ACR and Bohan/Peter criteria did not incorporate the presence of ani-MDA5 Abs as a criterion. Thus, approximately 30% of ADM patients may have been neglected by these two criteria [19]. Previous studies have emphasized the risk factors for anti-MDA5^+^-DM with RPILD. However, other anti-MDA5 antibody-associated diseases, such as subsets not fulfilling either criteria, who may or may not develop RPILD subsequently, have not been investigated. Therefore, we designed the present study to clarify different baseline conditions, clinical features, and laboratory manifestations in anti-MDA5^+^ patients with or without RPILD.

## 2. Materials and Methods

### 2.1. Patients and Study Design

We included all anti-MDA5^+^ patients and stratified them according to 2017 ACR/EULAR, Sontheimer’s or 1975 Bohan/Peter criteria into those with DM/RPILD and those without DM but with or without RPILD in Taipei Veterans General Hospital (TVGH) from 2018 to 2022 (Supplement Figure S1) [20,21,22]. The anti-MDA5 Abs or other myositis-specific Abs were measured by the kits that are commercially available (EUROIMMUN (South East Asia) Pte. Ltd., Taipei, Taiwan) in routine outpatient or inpatient practice. All patients’ medical records were retrospectively reviewed. A total of 73 patients were found anti-MDA5^+^. Two of them were excluded because they only visited the outpatient clinic once without any pertinent data. Among the 71 included patients, 39 (55%) were classified as DM. The baseline data, clinical features, laboratory data, lung imaging, concurrent myositis Abs, concomitant malignancies, and a presence or absence of other CTDs (rheumatoid arthritis (RA), systemic lupus erythematosus (SLE), Sjögren syndrome, autoimmune hepatitis (AIH), mixed connective tissue disease (MCTD), systemic sclerosis (SSc), adult onset Still’s disease (AOSD), ankylosing spondylitis (AS), or immunoglobulin G4-related disease (IgG4-RD)) were compared between anti-MDA5^+^ patients with and without RPILD. Patients who were documented to have concurrent malignancies or other CTD must have been ILD-free before anti-MDA5 Abs were detected. All patients with CTD must have been regularly followed up in this hospital for at least 3 months.

ILD was identified through plain radiography and/or high-resolution computerized tomography (HRCT) of the chest. Those who were initially suspected to have ILD via a chest X-ray underwent subsequent HRCT scanning to confirm the diagnosis as our standard procedures in routine clinical practice. The HRCT images were independently and blindly reassessed by two radiologists after inclusion into the study protocol, ensuring inter-observer agreement. Individuals who developed progressive dyspnea, hypoxemia, and interstitial lesions as demonstrated on the imaging studies within 3 months from the onset of respiratory symptoms were defined as having RPILD [23]. A presence of interstitial pneumonia with autoimmune features (IPAF) must meet all *a priori* requirements and have at least one feature retrieved from at least two of the clinical, serological and morphological domains [24]. This investigation has been approved by the Institutional Review Board of the TVGH (IRB-No: 2021–06-021BC, 3 September 2021).

### 2.2. Statistical Analyses

The features of anti-MDA5^+^ patients with and without RPILD were compared by Student’s *t*-test. Fisher’s exact test was used to evaluate the differences in frequencies for elevated serum aspartate aminotransferase (AST, normal 10–36 U/L), lactate dehydrogenase (LDH, normal 140–280 U/L in adults), creatine kinase (CK, nomal 30–170 U/L), myoglobulin (normal 5–70 μg/L), C-reactive protein (CRP, normal < 0.5 mg/dL), erythrocyte sedimentation rate (ESR, normal < 20 mm/h), albumin (Alb, normal 3.7–5.4 gm/dL), carcinoembryonic antigen (CEA, normal < 5 μg/L), cancer antigen 15-3 (CA-153, normal ≤ 30 U/mL), IgE (normal < 180 IU/mL) and ferritin (normal 24–336 ng/mL) levels. *p* < 0.05 was considered statistically significant in two-tailed tests. Univariate and multivariate logistic regression analyses were used to calculate the odds ratio (OR) as well as 95% confidential interval (CI) for RPILD and death in anti-MDA5^+^-related diseases. Statistical Package for Social Science (SPSS) software V.25 (IBM Corp., Armonk, NY, USA) was used for the statistical descriptions and analyses.

## 3. Results

### 3.1. Clinical and Laboratory Features of Anti-MDA5^+^-Patients

A total of 71 anti-MDA5^+^-patients were included from January 2018 to August 2022. The basic characteristics of these patients are summarized in Table 1. In total, 25 (35.2%) were male and 46 (64.8%) were female. The mean age at inclusion was 63-year-old. As shown in Supplement Figure S1, 39 (55%) were recognized to have DM, according to the 2017 EULAR/ACR, 1975 Bohan-Peter or Sontheimer’s criteria, among whom 22 (31.0%) eventually developed RPILD. On the other hand, among the rest of the 32 patients that did not have DM, 11 (15.5%) also developed RPILD. Among these 33 (46.5%) patients with RPILD, 20 (28.1%) were categorized as nonspecific interstitial pneumonia (NSIP) and 13 (18.3%) were categorized as usual interstitial pneumonia (UIP). Uniquely, four (5.6%) of those without DM were also classified as having interstitial pneumonia with autoimmune feature (IPAF). In total, 27 (38.0%) were recognized to have other CTDs (overlapped with or existing without DM, as listed in Supplement Table S1) and 15 (21.1%) were co-morbid with malignancy (Supplement Table S2). The CT pattern and pulmonary function data of 33 RPILD patients are summarized in Supplement Table S3.

Although being insignificantly different, the anti-MDA5^+^ RPILD group tended to be older with a higher presence of serum AST, LDH, CRP, ESR, CA-153, CEA, IgE, and/or ferritin, but lower albumin, myoglobin, and CK levels than the non-RPILD counterparts.

### 3.2. Basic Laboratory Data, Anti-SAE Antibodies, Anti-Ro52 Antibodies and the Mortality

Compared with the averages in the parent cohort (N = 71), patients with higher LDH (508.4 ± 237.5 U/L, *p* = 0.009), CRP (8.78 ± 8.97 mg/dL, *p* = 0.001), CEA (15.2 ± 8.6 ng/mL, *p* = 0.001) and ferritin (3178.2 ± 2147.4 ng/mL, *p* ≤ 0.001) but lower albumin (2.58 ± 0.43 g/dL, *p* ≤ 0.001) were significantly associated with mortality.

Anti-SAE Abs were present in 32 (45.1%) patients, and anti-Ro52 Abs were present in 30 (42.3%) patients. Anti-SAE Abs occurred more frequently in anti-MDA5^+^-non-RPILD patients (71.9% vs. 28.1%). Furthermore, compared with the averages in the parent cohort, the LDH (360.8 ± 127.3 U/L, *p* = 0.043) and albumin levels (3.57 ± 0.84 g/dL, *p* = 0.037) were significantly associated with mortality, whereas the LDH level (411.8 ± 179.7 U/L) was significantly lower and the albumin level (3.32 ± 0.79 g/dL) was significantly higher in anti-MDA5^+^ and anti-SAE^+^ double-positive patients. The AST, myoglobulin, ESR, CA-153, CEA, and ferritin levels were lower in the anti-SAE^+^ cohort than in the parent cohort. On the contrary, aside from the albumin, CRP and IgE levels, other laboratory results were higher in the anti-MDA5^+^ and anti-Ro52^+^ double-positive cohort than in the parent cohort.

### 3.3. Subgroup Analysis: Stratification by the Positivity of Anti-SAE Antibodies

To further clarify the role of anti-SAE in RPILD and mortality, we stratified the patients into positive or negative anti-SAE antibody groups. As shown in Table 2, the risk factor of developing RPILD in the negative anti-SAE group was elevated CRP (OR = 17.111, *p* = 0.04), CEA (OR = 35, *p* = 0.026) and ferritin (OR = 19, *p* = 0.032). In contrast, none of these odds ratios were seen in the anti-SAE positive group.

As regards mortality (Table 3), the causes of death were generally similar between the anti-SAE-positive and -negative groups. In both groups, deaths were primarily attributed to rapid progressive interstitial lung disease with secondary infection. The mortality rate in patients with positive anti-SAE and RPILD was 33.3% (3/32), and the mortality rate in those without anti-SAE but with RPILD was 60.9% (14/39). There were three anti-SAE (+) patients who succumbed during the study period. They were male, aged 74, 79, and 59 years, respectively. All of them developed RPILD with UIP pattern. The first patient died of pneumonia with acute respiratory failure. Notably, this patient also experienced pneumothorax during the hospitalization. The second one passed away due to aspiration pneumonia. The third succumbed ultimately to aspiration pneumonia with secondary respiratory failure despite previous tracheostomy. Additionally, he also experienced pneumothorax during his last hospitalization.

RPILD was a major risk factor for mortality in both groups; however, the likelihood ratio (LR) was much lower in the anti-SAE (+) cohort (8.455, *p* = 0.004) than in their negative counterparts (20.131, *p* < 0.001). In addition, more risk factors were identified in the antibody-negative group, including elevated CRP (LR = 8.238, *p* = 0.04), CEA (LR = 7.664, *p* = 0.06), ferritin (LR = 5.868, *p* = 0.015) and AST (LR = 8.289, *p* = 0.004) and lower albumin (LR = 8.388, *p* = 0.004) levels. In the anti-SAE (+) group, not only were fewer risk factors identified (only elevated CRP (LR = 4.439, *p* = 0.034) and lower albumin (LR = 5.194. *p* = 0.023)), but the LR was also lower than in the anti-SAE (−) counterparts. Respiratory failure was less likely to occur in the anti-SAE (+) group, with only 29.1% of them developing RPILD.

### 3.4. Subgroup Analysis: Stratification by Diagnosis of DM or Not

As shown in Table 4, the mean ages in the non-DM and anti-MDA5-positive group tended to be older than those in the DM with anti-MDA5 group (64.7 years old vs. 62.4 years, *p* = 0.480), although not significantly so. On the other hand, the LDH and ferritin levels were significantly lower in the non-DM with MDA5 group (LDH: 344.16 vs. 468.43, *p* = 0.04, ferritin: 959.89 vs. 2179.56, *p* = 0.033). The positivity rates of anti-Ro52 or anti-SAE Abs were also different between the two group, with anti-Ro52 being more prevalent in the DM group (73.3% vs. 26.7%, *p* = 0.009) and anti-SAE Abs being more prevalent in the non-DM group (75% vs. 25%, *p* < 0.001). Furthermore, the mortality rate was higher in the DM group (OR = 5.413, *p* = 0.012), who also tended to fewer less anti-SAE and more anti-Ro52 Abs.

### 3.5. Kaplan–Meier Survival Analysis of the Parent Anti-MDA5 Cohort, Anti-SAE^+^ and Anti-Ro52^+^ Groups

**Figure 1:**
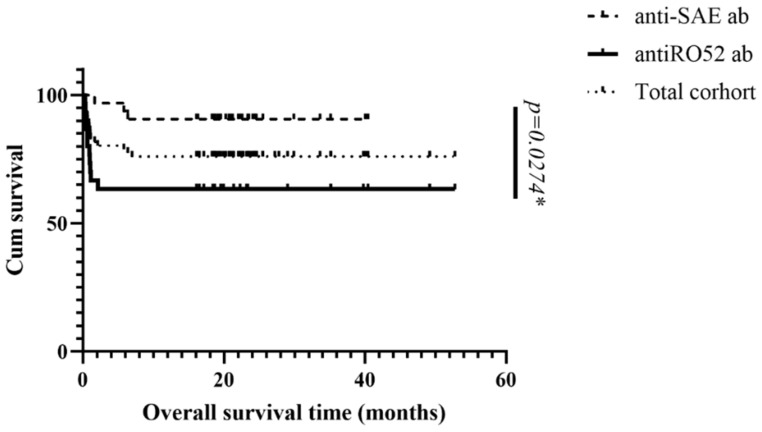
Kaplan–Meier survival curve for the parent cohort, patients with anti-SAE Abs, and patients with anti-Ro52 Abs; * significant.

**Figure 2:**
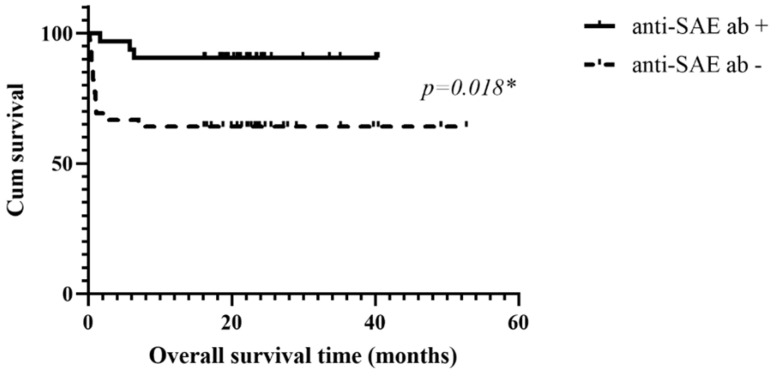
Kaplan–Meier survival curve for the patients with and without anti-SAE Abs; * significant.

**Figure 3:**
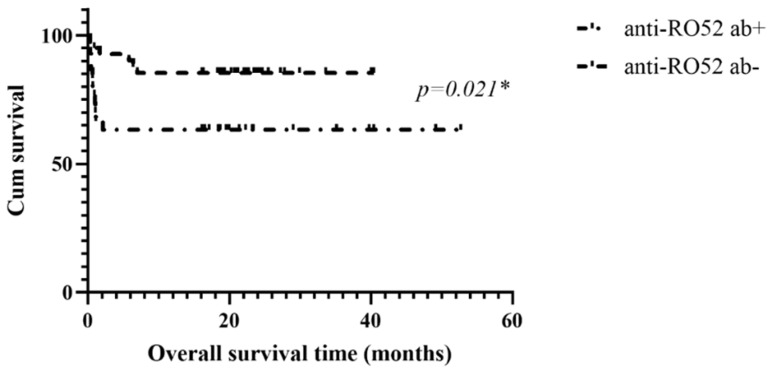
Kaplan–Meier survival curve for the patients with and without anti-Ro52 Abs; * significant.

The survival curves for the three groups are presented in Figure 1. The median survival months for patients with anti-SAE antibodies, the parent cohort, and patients with anti-Ro52 antibodies were 47.7, 40.5, and 38.7, respectively (*p* = 0.0274). Compared to the patients without anti-SAE Abs, those with anti-SAE Abs showed a significantly longer median survival time (47.7 vs. 42.4 months, *p* = 0.018, Figure 2). Conversely, patients with anti-Ro52 Abs showed a significantly shorter median survival time than their negative counterparts (38.7 months vs. 42.4 months, *p* = 0.021, Figure 3).

### 3.6. Univariate and Multivariate Logistic Regression Analyses for Risk Factors of RPILD in Anti-MDA5^+^ Patients

Univariate and multivariate analyses were performed to calculate the risk factors for RPILD in anti-MDA5^+^ patients (Table 5). As analyzed by univariate regression, anti-Ro52 Abs were an independent risk factor (OR = 3.331 CI = 1.25–8.91, *p* = 0.017), whereas anti-SAE Abs were conversely a significant protective factor (OR = 0.245 CI = 1.25–8.91, *p* = 0.017) for RPILD. Furthermore, according to multivariate regression, anti-SAE Abs still served as a protective factor from RPILD (OR = 0.058, CI = 0.004–0.829, *p* = 0.036).

### 3.7. Subgroup Analysis: Odds Ratio for RPILD and Mortality

**Figure 4:**
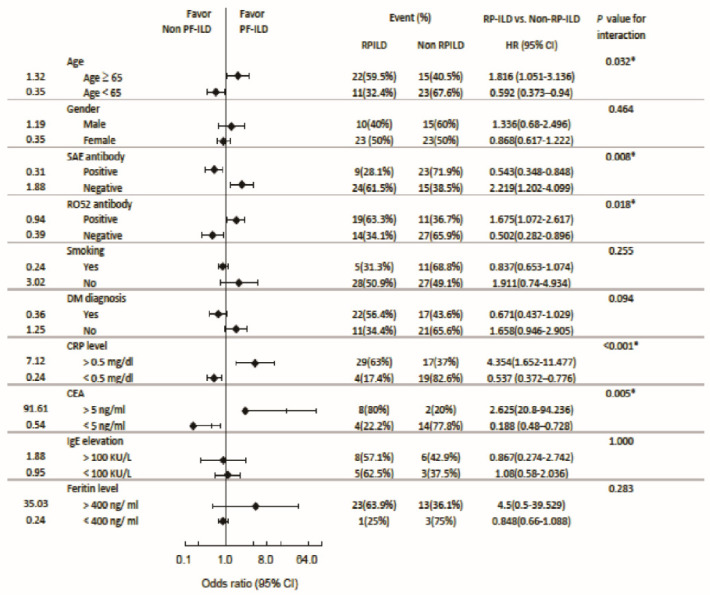
Forest plot to show odds ratios for RPILD or non-RPILD. * *p* < 0.05.

The OR of various factors for RPILD are shown in Figure 4. An age ≥ 65 years was a risk factor (OR = 1.816, CI = 1.051–3.136, *p* = 0.032). Other risk factors for RPILD included anti-Ro-52^+^ Abs (OR = 1.676, CI = 1.072–2.617, *p* = 0.018), elevated CRP (OR = 4.354, CI = 1.652–11.477, *p* < 0.001) and CEA (OR = 2.625, CI = 20.800–94.236, *p* = 0.005) levels, as well as an absence of anti-SAE Abs (OR = 2.219, CI = 1.202–4.099, *p* = 0.008). On the other hand, anti-SAE Abs served as a protective factor from RPILD (OR = 0.543, CI = 0.348–0.848, *p* = 0.008). Additional protective factors included an absence of anti-Ro52-Abs (OR = 0.502, CI = 0.282–0.896, *p* = 0.018), age below 65 years (OR = 0.592, CI = 0.373–0.940, *p* = 0.032), and normal CRP (OR = 0.537, CI = 0.372–0.776, *p* < 0.001) and CEA (OR = 0.188, CI = 0.48–0.728, *p* = 0.005) levels.

**Figure 5:**
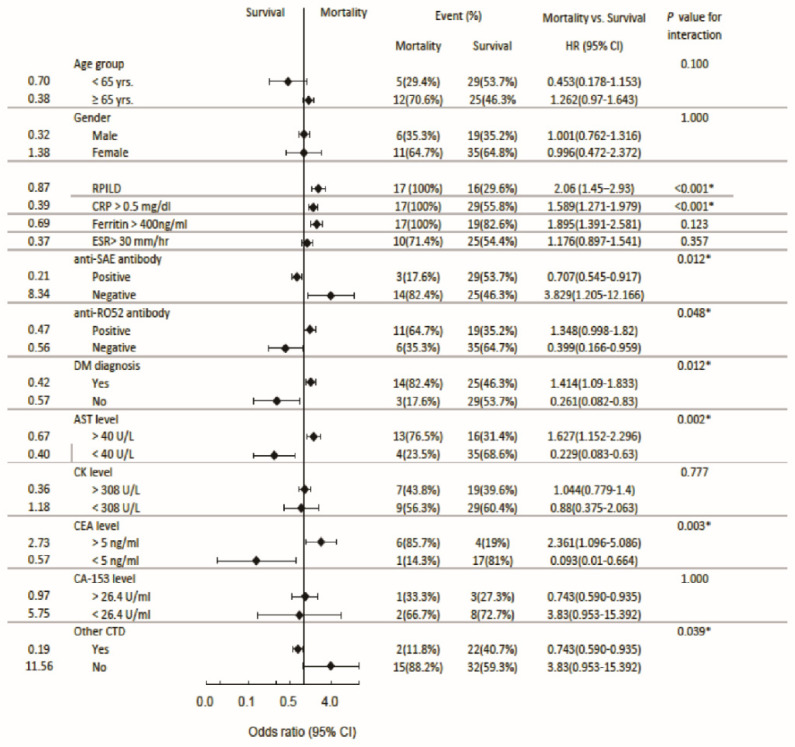
Forest plot to show odds ratios for mortality in RPILD patients. * *p* < 0.05.

Figure 5 shows the risk factors for mortality. Although not significantly, older patients tended to have a higher mortality rate than younger patients. Anti-SAE Abs not only served as a protective factor from RPILD, but also from mortality (OR = 0.707, CI = 0.545, *p* = 0.012). A presence of anti-SAE Abs and the absence of anti-Ro-52-Abs were prone to exclusion of DM. Normal AST and CEA in anti-MDA5^+^ patients comorbid with other CTDs posed a protective factor from mortality.

## 4. Discussion

Anti-MDA5 Abs are notorious for causing RPILD and high mortality rates in DM patients [25]. Various therapeutic regimens have been attempted in order to lower the high death rate [26,27,28,29], but there is still a lack of randomized controlled trials or large prospective studies to provide evidence-based treatments. Because of a limited treatment and a poor prognosis, a high index of suspicion with earlier and adequate interventions is important to prevent further morbidity and mortality. Moreover, identifying the coexisting parameters influencing MDA5 antibody’s favoring of the development of RPILD is essential as well.

Numerous studies dealing with the risk factors for RPILD in DM patients have revealed that old age, elevated LDH, ferritin, CEA, CA-153, and CRP, the presence of anti-Ro52 antibodies, a high titer of anti-MDA5 antibodies, as well as a short disease duration of less than 3 months are the key risk factors [30,31,32]. Furthermore, a prediction model based on LDH, age and white cell count (FLAW model) has also been proposed to stratify the risks [33]. In the present study, senility, anti-Ro52 antibodies, and high CRP and CEA levels were found to be associated with the development of RPILD, which is compatible with previous research. As regards mortality, our findings are in accordance with those of other studies, i.e., high CRP, ferritin, AST, and CEA levels, as well as existing RPILD, might contribute to mortality. Moreover, definite DM might also increase the mortality. Conversely, anti-MDA5^+^ diseases other than DM might otherwise decrease the mortality. Compared with these non-DM anti-MDA5^+^ individuals, most DM patients with anti-MDA5 Abs (73%) showed more anti-Ro52 and fewer anti-SAE antibodies.

To the best of our knowledge, there has been no study dealing with the relationship between anti-SAE Abs and RPILD. Encouragingly, the present study demonstrated that anti-SAE Abs were not only a protective factor from anti-MDA5^+^ RPILD, but also a favorable parameter for getting rid of mortality. Anti-SAE^+^- patients have showed cutaneous lesions such as Göttron’s papules, violaceus rash, or the absence of overt muscle involvement [17,18,34]. Other manifestations including dysphagia, arthritis, constitutional symptoms as well as ILD were heterogenous, as reported in different studies [35,36].

Except for anti-Ro52 Abs, it is unclear whether two myositis-specific antibodies (MSAs) will interact with each other when coexisting. Anti-Ro52 antibodies have been strongly correlated with anti-synthetase^+^-ILD and anti-MDA5^+^-ILD [37]. On the other hand, Ge et al. found that one-third of anti-SAE^+^-patients can simultaneously carry other MSAs uneventfully. Furthermore, the incidence of ILD in DM associated with anti-SAE Abs was higher in an Asian than in a European cohort [18].

The average age of our patients (63.4 years) was older than that reported in most of the other DM studies. This is consistent with a previous theory that the DM-onset age in the anti-SAE^+^-group is significantly older than that in the antibody-negative group [18]. It also suggests that anti-SAE does have some protective effect in delaying the onset of autoimmune lung injury, although its mechanism is still not well clarified. On the other hand, almost half of our anti-MDA5^+^ patients (45.2%) also showed anti-SAE Abs, the prevalence rate being higher than that shown in the previous Asian report. The prevalence of anti-SAE antibodies can vary among different populations and cohorts, influenced by a multitude of factors, including genetic, environmental, and lifestyle components such as diet, smoking, and exposure to infectious agents. Notably, our patient population displayed an older age profile compared to other studies on anti-SAE antibodies, suggesting that age might be a primary contributing factor to the higher prevalence observed in our cohort.

According to our results, anti-SAE Abs might be regarded as a protective factor from both RPILD and mortality when they coexist with anti-MDA5 Abs. A previous study conducted by Ge et al. has shown that anti-SAE^+^-DM patients have a disease activity lower than their anti-SAE^-^ counterparts. Since anti-SAE Abs tend to have this protective effect, anti-MDA5^+^ RPILD may also be guarded by anti-SAE Abs, regardless of the definite presence of DM or not. Besides this, anti-SAE^+^ -patients mainly present with typical cutaneous lesions of DM, which may alert the caregivers to investigate and intervene in patients earlier, preventing possible ILD progression or mortality.

Of note, our study has also revealed the heterogeneous spectrum of anti-MDA5 antibody-associated diseases. As distinct from the findings of a non-Asian study conducted by Cavagna L et al. [38], 24 (33.8%) of our patients showed other autoimmune diseases. Despite not being associated with RPILD, concurrent CTDs were protective factors from mortality, which might have resulted from the prompt delivery of immunosuppressants for these CTDs. Although only four of our patients were diagnosed with anti-MDA5^+^-IPAF, none of the patients had concomitant anti-SAE Abs.

Despite the pertinent findings demonstrated in this study, there were several limitations in it. Firstly, our autoantibody detections were totally dependent on the EUROLINE test kit; hence, the false positive and negative rates cannot be ignored. In addition, because it is a retrospective study, some medical records are inevitably incomplete. These may include patients’ physical examination (PE) records, including data on Göttron’s sign, heliotrope sign or mechanic’s hands, and because of this we were unable to analyze the correlation between PE and RPILD or mortality. Nevertheless, with so many missing data, a high CEA level was still significantly associated with RPILD and mortality. This has reinforced the hypothesis that CEA activates macrophage or Kupffer cells to cause further pulmonary inflammation [31]. In addition, anti-SAE Abs might serve as a protective factor from RPILD or mortality in anti-MDA5^+^ RPILD patients, but we will require more functional assays to verify this and explore the underlying mechanism. Finally, this is an Asian-based study. Whether its results can apply to different ethnicities is questionable, leaving a large space to be filled in.

In conclusion, the present investigation has not only confirmed previous risk factors in anti-MDA5^+^-DM-associated RPILD, such as old age, ferritin, CRP, AST, CEA and anti-Ro52 Abs, but it has also identified anti-SAE Abs as a protective factor in Asian anti-MDA5 Abs-associated diseases, especially in those concurring with RPILDs with a high mortality rate.

## Figures and Tables

**Table 1:** Baseline characteristics of 71 patients with anti-MDA5 antibodies.

	Total Patients (*n* = 71)	RPILD * (*n* = 33)	Non RPILD (*n* = 38)	Concurrent Anti-SAE Abs (*n* = 32)	Concurrent Anti-Ro52 Abs (*n* = 30)	Mortality (*n* = 17)
Male (%)	25 (35.2%)	10 (30.3%)	15 (39.5%)	14 (43.8%)	6 (20%)	6 (35.3%)
Female (%)	46 (64.8%)	23 (69.7%)	23 (60.5%)	18 (56.3%)	24 (80%)	11 (64.7%)
Mean age (S.D. **)	63.4 (13.9)	65.5 (11.1) (*p* = 0.239)	61.6 (15.8) (*p* = 0.238)	64.5 (14.2) (*p* = 0.546)	60.9 (13.5) (*p* = 0.192)	67.1 (9.4) (*p* = 0.21)
DM/PM (%)	39 (55%)	22 (56.4%)	17 (43.6%)	8 (25%)	22 (73.3%)	14 (82.4%)
AST (S.D.**) U/L	79.8 (105.9)	97.9 (126.5)	63.6 (82.0)	61.0 (89.5) (*p* = 0.194)	106.1 (132.5) (*p* = 0.086)	107.9 (82.8) (*p* = 0.209)
LDH (S.D. **) U/L	411.8 (179.7)	427.5 (205.7)	397.0 (152.9)	360.8 (127.3) (***p* = 0.043**)	448.1 (218.7) (*p* = 0.139)	508.3 (237.5) (***p* = 0.009**)
CK (S.D. **), U/L	928.1 (2318.8)	504.2 (847.4)	1279.3 (3015.2)	832.4 (2931.7) (*p* = 0.926)	959.3 (1732.9) (*p* = 0.059)	629.8 (1084.8) (*p* = 0.234)
Myoglobulin (S.D. **), ng/mL	574.5 (851.6)	548.1 (819.1)	606.8 (938. 9)	157.6 (122.4) (*p* = 0.11)	582.4 (854.6) (*p* = 0.968)	448.3 (693.6) (*p* = 0.602)
CRP (S.D.**) mg/dL	4.17 (6.80)	6.03 (8.18)	2.46 (4.74)	4.25 (6.18) (*p* = 0.937)	3.36 (6.56) (*p* = 0.405)	8.78 (8.97) (***p* = 0.001**)
ESR (S.D.**) mm/h	50.0 (35.9)	56.2 (36.6)	44.5 (34.9)	47.5 (35.3) (*p* = 0.634)	50.4 (32.8) (*p* = 0.94)	61.3 (34.4) (*p* = 0.18)
Albumin (S.D. **), g/dL	3.32 (0.79)	3.11 (0.82)	3.56 (0.69)	3.57 (0.84) (***p* = 0.037**)	3.10 (0.74) (*p* = 0.098)	2.58 (0.43) (***p* = <0.001**)
CA-153(S.D. **), U/mL	19.9 (17.8)	32.9 (18.8)	10.1 (9.2)	10.5 (10.5) (*p* = 0.087)	26.1 (7.8) (*p* = 0.517)	29.0 (12.8) (*p* = 0.339)
CEA (S.D. **), ng/mL	7.8 (7.4)	12.5 (8.4)	4.2 (3.9)	6.9 (6.7) (*p* = 0.569)	11.0 (9.8) (*p* = 0.239)	15.2 (8.6) (***p* = 0.001**)
IgE (S.D. **), KU/L	260.7 (306.8)	310.9 (378.1)	188.1 (150.3)	298.1 (402.8) (*p* = 0.58)	155.1 (215.9) (*p* = 0.335)	392.0 (491.9) (*p* = 0.176)
Ferritin (S.D. **) ng/mL	1722.2 (1920.2)	2449.8 (2152.8)	630.8 (602.0)	1063.3 (1338.1) (*p* = 0.076)	2245.4 (1982.7) (*p* = 0.120)	3178.2 (2147.4) (***p* < 0.001**)
Smoking (%)	16 (22.5%)	5 (15.2%)	11 (28.9%)	9 (56.3%)	4 (25%)	2 (11.8%)
Malignancy (%)	15 (21.1%)	11 (73.3%)	4 (26.7%)	5 (33.3%)	2 (13.3%)	6 (35.3%)
Other CTD *** (%)	27 (38%)	10 (41.7%)	14 (58.3%)	13 (54.2%)	13 (54.2%)	2 (11.8%)
Anti-SAE antibody (%)	32 (45.1%)	9 (28.1%)	23 (71.9%)		5 (15.6%)	3 (17.6%)
Anti-Ro52 antibody (%)	30 (42.3%)	19 (63.3%)	11 (36.7%)	5 (15.6%)		11 (64.7%)
Mortality (%)	17 (23.9%)	17 (100%)	0 (0)	3 (17.6%)	11 (64.7%)	

The presence of Abs was detected by Euroimmun kit, SAE and Ro52 Abs being found to have the strongest negative or positive correlations with ILD. Abs other than anti-SAE or Ro52 were rarely encountered, and so are not shown in this table. All comparisons were made between the individual subgroups and the total patients (parent cohort, N = 71) with *p* values < 0.05 as significant. * Rapidly progressive interstitial lung disease. ** S.D., standard deviation. *** CTD, connective tissue disease: RA, SLE, Sjögren, AIH, MCTD, SSc, AOSD, AS, IgG4-RD. Missing data: AST, LDH, myoglobulin, CRP, ESR, albumin, CA-153, CEA, IgE, ferritin. The *p* values that are significant are shown in bold. Normal ranges of albumin, ESR, CRP, AST, LDH, CA-153, CEA, IgE, myoglobulin and ferritin are listed in Section 2.

**Table 2:** Subgroup analysis of RPILD in anti-SAE antibody-positive and -negative groups.

	Anti-SAE-Positive (N = 32)				Anti-SAE-Negative (N = 39)			
	RP-ILD (N = 9, 28.1%)	Non-RP-ILD (N = 23, 71.9%)	*p*-Value	OR *** (C.I^.#^)	RP-ILD (N = 23, 59%)	Non-RP-ILD (N = 16, 41%)	*p*-Value	OR *** (C.I. ^#^)
LDH (S.D. **) U/L	376.22 (112.3)	353.9 (135.6)	0.670	0.441 (*p* = 0.442, 0.057–3.421)	456.43 (230.4)	439.94 (165.1)	0.807	3.385 (*p* = 0.55, 0.279–41.087)
CRP (S.D. **) mg/dL	8.23 (8.9)	2.547 (3.7)	**0.018** *	2.667 (*p* = 0.427, 0.521–13.655)	5.40 (8.04)	2.26 (5.9)	0.192	**17.111 (*p* = 0.04,** 1.832–159.802 *)
Albumin (S.D. **), g/dL	3.31 (1.1)	3.67 (0.75)	0.358	1.905 (*p* = 0.659, 0.321–11.312)	3.05 (0.8)	3.33 (0.5)	0.352	1.583 (*p* = 0.634, 0.23–10.904)
CEA (S.D. **), ng/mL	11.45 (9.6)	5.15 (4.7)	0.116	3 (*p* = 0.505, 0.14–64.262)	13.02 (8.4)	2.67 (1.6)	**0.012** *	**35 (*p* = 0.026,** 1.743–702.993 *)
Ferritin (S.D. **) ng/mL	2581.25 (2103.6)	557.32 (343.2)	**0.004** *	0.597 (*p* = 1.00, 0.773–1.087)	2423.49 (2215.2)	851.15 (1137.4)	0.186	**19 (*p* = 0.032,** 1.146–314.971*)
CK (S.D. **), U/L	157.29 (99.6)	1068.7 (3395.8)	0.49	0.489 (*p* = 0.662, 0.76–3.145)	620.86 (972.5)	1492.6 (2436.2)	0.144	0.791 (*p* = 0.749, 0.211–2.972)
AST (S.D. **) U/L	48.38 (38.1)	65.86 (103.1)	0.647	1.067 (*p* = 0.647, 0.161–7.056)	118.43 (143.2)	58.19 (38.8)	0.111	2.411 (*p* = 0.209, 0.652–8.92)
Myoglobulin (S.D. **), ng/mL	92.5 (130.8)	183.68 (123.4)	0.423	0.25 (*p* = 0.524, 0.07–8.56)	6 (66.7%)	3 (33.3%)	0.439	0.667 (*p* = 0.646, 0.047–9.472)
ESR (S.D. **) mm/h	48.14 (29.8)	47.3 (37.8)	0.958	1.333 (*p* = 0.546, 0.235–7.556)	58.25 (39.8)	42.38 (30.4)	0.231	1.333 (*p* = 0.465, 0.235–7.556)
IgE (S.D. **) KU/L	468.96 (567.1)	155.72 (117.2)	0.215	0.75 (*p* = 0.652, 0.064–8.834)	212.1 (179.7)	252.91 (215.8)	0.756	0.75 (*p* = 0.721, 0.064–8.834)
anti-Ro52 antibodies (+)	2 (22.2%)	7 (77.8%)	-	0.653 (*p* = 0.501, 0.107–3.971)	2 (22.2%)	7 (77.8%)	-	0.653 (*p* = 0.179, 0.107–3.971)
Smoking	2 (22.2%)	7 (77.8%)	-	0.653 (*p* = 0.501, 0.107–3.971)	2 (22.2%)	7 (77.8%)	-	0.653 (*p* = 0.415, 0.107–3.971)

The *p*-values that are significant (*) are shown in bold. ** S.D., standard deviation; *** OR, odds ratio; ^#^ C.I, confidence interval.

**Table 3:** Subgroup analysis of mortality in anti-SAE-positive and -negative groups.

	Anti-SAE-Positive Anti-SAE-Negative							
	Mortality (N = 3, 9.4%)	Non-Mortality (N = 29, 90.6%)	*p*-Value	LR (C.I.)	Mortality (N = 14, 35.9%)	Non-Mortality (N = 25, 64.1%)	*p*-Value	LR (C.I.)
RPILD	3 (33.3%)	6(66.7%)	-	**8.455 (*p* = 0.004** * 0.101–0.422)	14 (60.9%)	9 (39.1%)	-	**20.131 (*p* < 0.001,** 0.213–0.607 *)
LDH (S.D. **) U/L	392.67 (183.8)	19 (86.4%)	0.656	1.311 (*p* = 0.252 0.645–0.972)	402.92 (245.9)	402.92 (163.4)	0.054	2.906 (*p* = 0.088, 0.752–1.018)
CRP (S.D. **) mg/dL	11.68 (7.4)	3.42 (5.6)	**0.025** *	**4.493 (*p* = 0.034** * 0.292–0.678)	8.16 (9.4)	1.84 (4.7)	**0.008** *	**8.236 (*p* = 0.04,** 0.52–0.89 *)
Albumin (S.D. **) g/dL	2.4 (0.529)	3.73 (0.8)	**0.007** *	**5.194 (*p* = 0.023** * 0.221–0.657)	2.62 (0.4)	3.54 (0.6)	**0.000** *	**8.388 *(p* = 0.004** * **,** **0.455–0.919)**
CEA (S.D. **) ng/mL	11.95 (12.4)	6.12 (5.8)	0.272	1.827 (*p* = 0.177 0.351–345.1)	16.5 (8.2)	4.19 (4)	**0.002** *	7.664 (*p* = 0.06 *, **0.132–0.84)**
Ferritin (S.D. **) ng/mL	3038.33 (2320.3)	607.53 (375.1)	0.001 *	0.43 (*p* = 0.512 0.789–1.08)	3208.14 (2200.4)	696.04 (789)	**0.002** *	5.868 (*p* = 0.015 *, **0.467–0.905)**
CK (S.D. **) U/L	91.33 (45.8)	925.04 (3104)	0.651	3.394 (*p* = 0.065 0.375–1.783)	754 (1175.3)	1129.9 (2054.4)	0.549	1.463 (*p* = 0.226, 0.586–9.291))
AST (S.D. **, U/L	53.67 (64.6)	61.88 (92.9)	0.884	0.145 (*p* = 0.704 0.128–21.732)	119.5 (83.5)	79.28 (129.8)	0.304	8.289 (*p* = 0.004 *, **1.648–49.137)**
Myoglobulin (S.D. **), ng/mL	124.3 (120.3)	183.9 (110.4)	0.184	2.969 (*p* = 0.085 0.028–1.997)	512.34 (723.1)	1133.4 (1219.3)	0.279	0.034 (*p* = 0.853, 0.118–13.24)
ESR (S.D. **) mm/h	86.5 (3.5)	44.4 (34.9)	0.106	2.776 (*p* = 0.096 0.319–1.722)	57.08 (35.6)	49.1 (37.9)	0.556	0.075 (*p* = 0.784, 0.278–5.454)
IgE (S.D. **) KU/L	1053.5 (369.8)	130.24 (106.6)	**0.000** *	2.055 (*p* = 0.152 0.15–0.455)	127.36 (149.8)	303.12 (172.6)	0.108	2.284 (*p* = 0.131, 0.008–2.181)
anti-Ro52 antibodies (+)	0 (0%)	9 (100%)	-	2.1 (*p* = 0.147 0.54–3.88)	0 (0%)	9 (100%)	-	2.076 (*p* = 0.15, 0.642–12.926)
Smoking (+)	1 (11.1%)	8 (88.9%)	-	0.043 (*p* = 0.836 0.104–16.55)	1 (11.1%)	8 (88.9%)	-	1.949 (*p* = 0.163, 0.026–2.269)

The *p*-values that are significant (*) are shown in bold. ** S.D., standard deviation.

**Table 4:** Subgroup analysis on the non-DM patients vs. DM patients, both with anti-MDA5 Abs.

	Non-DM with Anti-MDA5	DM with Anti-MDA5	*p*-Value
Age, y/o	64.7 (13.9)	62.4 (13.9)	0.480
AST (S.D.), U/L	63.83 (89.1)	91.64 (116.6)	0.287
LDH (S.D.), U/L	344.16 (133.9))	468.43 (194.7)	**0.04** *
Myoglobulin (S.D.), ng/mL	212.67 (163.4)	729.59 (981.5)	0.223
CRP (S.D.), mg/dL	4.19 (6.9)	4.15 (6.8)	0.981
ESR (S.D.), mm/h	51.12 (37.6)	49.1 (35.0)	0.833
Ferritin (S.D.), ng/mL	959.89 (1427.2)	2179.56 (2054.8)	**0.033** *
CA-153 (S.D.), U/mL	21.4 (26.3)	19.08 (12.9)	0.826
CEA (S.D.), ng/mL	7.75 (6.47)	7.78 (8.1)	0.99
IgE (S.D.), KU/L	296.5 (369.7)	208.9 (192.2)	0.524
Alb (S.D.), g/dL	3.46 (0.82)	3.22 (0.76)	0.278
CK (S.D.), U/L	755.8 (2839)	1039.2 (1772.1)	0.657
Anti-RO52 antibody	8 (26.7%)	22 (73.3%)	**0.009** *
Anti-SAE antibody	24 (75%)	8 (25%)	**<0.001** *
RPILD	11 (33.3%)	22 (66.7%)	0.094
Mortality ^#^	3 (17.6%)	14(82.4%)	**0.012** *

The *p*-values that are significant (*) are shown in bold. ^#^ Compared to non-DM with anti-MDA5+, the odds ratio for mortality in DM with anti-MDA5+ is 5.413 (C.I. = 1.394–21.025).

**Table 5:** Univariate and multivariate logistic regression analysis for the risk factors with RPILD in anti-MDA5^+^ patients.

Variable	Univariate			Multivariate		
	OR	95% CI of OR	*p* Value	OR	95% CI of OR	*p* Value
Female	1.500	0.559–4.025	0.421	0.494	0.057–4.311	0.523
High AST *	2.267	0.850–6.045	0.102	3.858	0.493–3.172	0.198
High LDH **	1.609	0.351–7.377	0.54	0.254	0.01–6.357	0.404
High Ferritin ***	5.308	0.5–56.391	0.166	10.713	0368–312.028	0.168
Ro52	3.331	1.245–8.91	**0.017** ^§^	2.198	0.116–41.489	0.599
SAE	0.245	0.9–0.668	**0.006** ^§^	0.058	0.004–0.829	**0.036** ^§^

OR = odds ratio; CI = confidence interval. * > 36 U/L; ** > 280 U/L; *** > 336 ng/mL; ^§^ s tatistically significant. Significant *p*-values are in bold.

## Data Availability

Data related to this study is unavailable due to privacy or ethical restrictions. The unavailability of the data is attributed to constraints imposed by ethical considerations or privacy regulations that prohibit the disclosure or sharing of specific information. This decision is made in accordance with ethical guidelines and data protection policies to ensure the confidentiality and privacy of individuals involved in the study.

## Supplementary Materials

The following supporting information can be downloaded at: https://www.mdpi.com/article/10.3390/jcm13030725/s1, Figure S1: The flow-chart for stratifying 71 anti-MDA5 positive patients; Table S1: Anti-MDA5^+^ patients with concurrent connective tissue disease; Table S2: Anti-MDA5^+^ patients concurrent with malignancies; Table S3: Thirty-three RP-ILD patients’ high-resolution CT pattern and associated pulmonary function test result.
