# Supplementary material for: Antibodies against Small Ubiquitin-like Modifier Activating Enzyme May Be a Protective Factor from Rapid Progressive Interstitial Lung Disease in Patients Bearing Antibodies against Melanoma Differentiation Associated Gene 5
Source: J Clin Med. 2024 Jan 26;13(3):725. doi: 10.3390/jcm13030725 (PMC10856636; doi:10.3390/jcm13030725)
Supplement: Supplementary file 1 [file jcm-13-00725-s001.zip › jcm-2802920-supplementary.pdf]

Supplement Table S1. Anti-MDA5^{+} patients with concurrent connective tissue disease

Connective tissue disease	Number (%)
Rheumatoid arthritis	6 (22%)
Systemic lupus erythematosus	4 (15%)
Sjögren syndrome	8 (30%)
Autoimmune hepatitis	3 (11%)
Mixed connective tissue disease	1 (4%)
Systemic sclerosis	2 (7%)
IgG4 related disease	1 (4%)
Adult-onset Still's disease	1 (4%)
Ankylosing spondylitis	1 (4%)
Total	27

Supplement Table S2. Anti-MDA5^{+} patients concurrent with malignancies

Malignancy	Number (%)
Breast cancer	3 (20%)
Prostate cancer	1 (7%)
Lung cancer	1 (7%)
Thyroid cancer	3 (20%)
Multiple myeloma	1 (7%)
Tongue cancer	2 (13%)
Hepatic cellular carcinoma	1 (7%)
Sarcoma	1 (7%)
Skin squamous cell carcinoma	1 (7%)
Myelodysplastic syndrome	1 (7%)
Total	15

Supplement table S3 Thirty-three RP-ILD patients' high-resolution CT pattern and associated pulmonary function test result.

Patient	Age	Gender	Radiologist 1 CT pattern	Radiologist 2 CT pattern	Previous CT or CXR (Date)	FVC (Date)	Previous FVC (Date)	DLCO	Previous DLCO	6MWT	Previous 6MWT	Mortality	Anti-SAE antibody positivity
1	67	F	NSIP	NSIP	CXR (202108)	N/A	89% (202108)	N/A	51% (202108)	N/A	N/A	Y	P
2	68	M	UIP	UIP	Chest CT (201908)	87% (202112)	91% (201908)	59.76% (202112)	64% (201908)	N/A	380m (201908)	N	P
3	74	M	UIP	UIP	Chest CT (202201)	71% (202201)	83% (202111)	50% (202201)	N/A	358m (202201)	N/A	Y	P
4	79	M	UIP	UIP	CXR (202111)	N/A	N/A	N/A	N/A	N/A	N/A	Y	P
5	70	F	UIP	UIP	CXR (202112)	N/A	80% (202112)	N/A	81% (202112)	N/A	N/A	N	P
6	74	F	NSIP	NSIP	CXR (202010)	N/A	N/A	N/A	N/A	N/A	N/A	Y	N
7	73	M	NSIP	NSIP	Chest CT (202005)	52% (202012)	64% (202005)	N/A	N/A	N/A	N/A	Y	N
8	65	F	NSIP	NSIP	CXR (201909)	N/A	N/A	N/A	N/A	N/A	N/A	Y	N

9	72	F	NSIP	NSIP	CXR (202203)	86% (202203)	N/A	36% (202203)	N/A	N/A	N/A	N	N
10	79	F	NSIP	NSIP	CXR (202201)	53% (202201)	N/A	N/A	N/A	126m (202201)	N/A	N	N
11	65	F	UIP	UIP	CXR (202207)	57% (202212)	59% (202206)	33% (202212)	N/A	N/A	394m (202206)	N	N
12	59	M	NSIP	NSIP	CXR (202201)	N/A	N/A	N/A	N/A	N/A	N/A	Y	P
13	66	M	NSIP	NSIP	CXR (202011)	78% (202011)	93% (202002)	50% (202011)	70% (202002)	N/A	359m (202002)	N	P
14	89	M	NSIP	NSIP	CXR (2019)	48% (2022)	59% (2018)	34% (2022)	44% (2018)	225m (2022)	N/A	N	N
15	62	F	UIP	UIP	CXR (2019)	85% (2019)	N/A	N/A	N/A	N/A	N/A	N	P
16	75	M	UIP	UIP	Chest CT (201912)	59% (202005)	N/A	26% (202005)	44% (2019)	N/A	N/A	N	N
17	65	F	NSIP	NSIP	CXR (202204)	58% (202212)	68% (20220428)	N/A	N/A	N/A	N/A	N	P
18	70	F	NSIP	NSIP	CXR (201907)	N/A	N/A	N/A	N/A	N/A	N/A	Y	N
19	49	F	NSIP	NSIP	Chest CT	37%	N/A	25%	NA	245m	N/A	Y	N

					(202011)	(202101)		(20210105)		(20210105)			
20	55	F	NSIP	NSIP	CXR (202009)	N/A	N/A	N/A	N/A	N/A	N/A	Y	N
21	74	F	NSIP	NSIP	CXR (202007)	N/A	N/A	N/A	N/A	N/A	N/A	Y	N
22	64	F	NSIP	NSIP	Chest CT (201910)	N/A	N/A	N/A	N/A	N/A	N/A	Y	N
23	49	F	NSIP	NSIP	Chest CT (201911)	38.3%i (202208)	N/A	N/A	N/A	N/A	N/A	Y	N
24	73	F	NSIP	NSIP	CXR (202203)	65% (202208)	79% 201705	53% (202208)	N/A	N/A	N/A	Y	N
25	59	F	UIP	UIP	CXR (202203)	55% (202111)	64% 202106	37% (202111)	57% (202106)	285m (202111)	347m (202106)	N	N
26	47	F	NSIP	NSIP	CXR (202008)	89% (202009)	92% 202009	70% (202009)	81% (202006)	N/A	N/A	N	N
27	65	F	UIP	UIP	CXR (202008)	67% (202011)	N/A	N/A	N/A	N/A	N/A	Y	N
28	77	M	UIP	UIP	Chest CT (202006)	N/A	N/A	N/A	N/A	N/A	N/A	Y	N
29	46	F	UIP	UIP	Chest CT (202006)	35% (202012)	44% 202007	15% (202012)	42% (202007)	175m (202012)	245m (202007)	N	N

30	75	F	UIP	UIP	Chest CT (201912)	N/A	N/A	N/A	N/A	N/A	N/A	Y	N
31	41	F	NSIP	NSIP	CXR (2019)	61% (2019)	65% 2018012	18% (2019)	19% (2018)	162m (2019)	301m (2018)	N	N
32	66	M	UIP	UIP	CXR (2019)	N/A	N/A	N/A	N/A	N/A	N/A	Y	N
33	50	F	NSIP	NSIP	CXR (2019)	63% (2020)	107%, (201903)	N/A	N/A	219m (2020)	482m (2019)	N	N

M, male; f, female; m, meter

NSIP, nonspecific interstitial pneumonia; UIP, usual interstitial pneumonia

FVC, forced vital capacity; DLCO, diffusing lung capacity for carbon monoxide; 6MWT, 6-minute walking test

CXR, Chest x ray; CT, computed tomography

N/A, not available

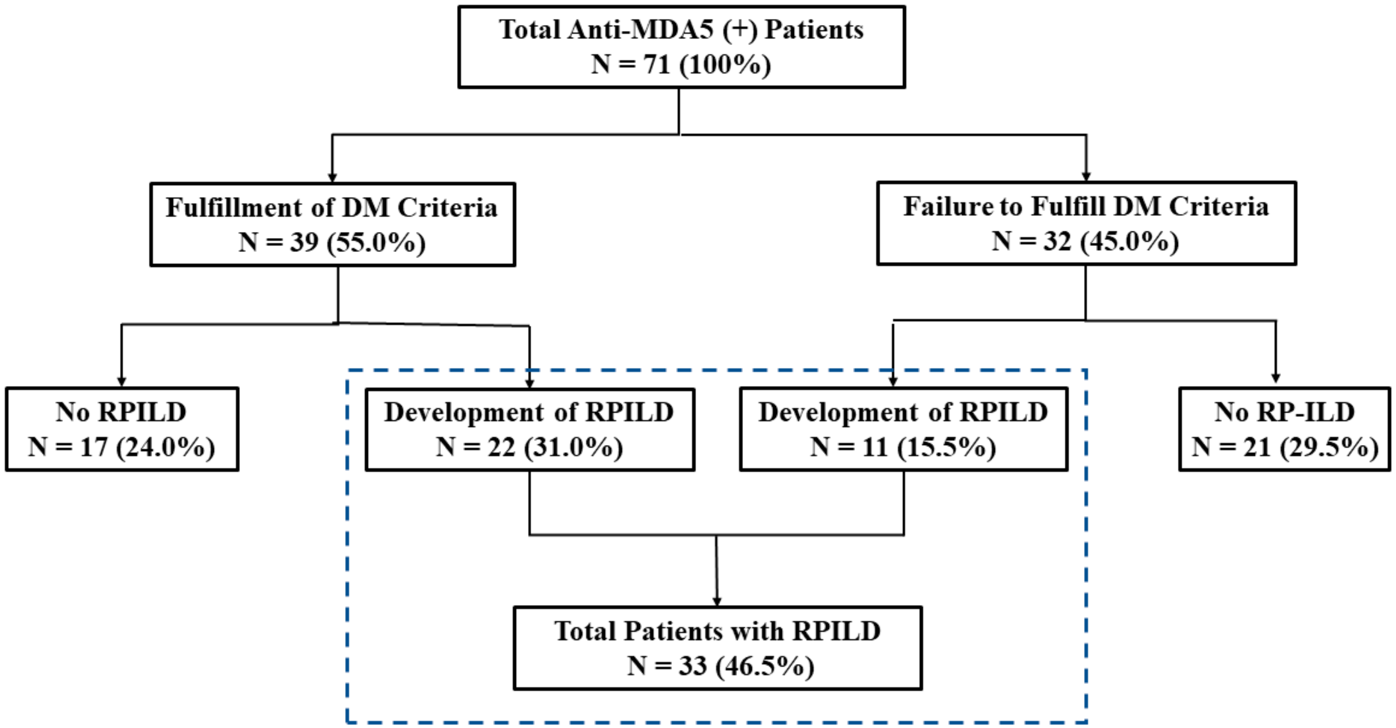

Supplement Figure S1. The flow-chart for stratifying 71 anti-MDA5 positive patients, 39 (55.0%) of whom fulfilled dermatomyositis criteria, 22 (31.0%) of whom developed rapidly progressive interstitial lung disease (RPILD), and 33 (46.5%) of whom developed RPILD.
